# Impact of rotavirus and hepatitis A virus by worldwide climatic changes during the period between 2000 and 2013

**DOI:** 10.6026/97320630015194

**Published:** 2019-03-15

**Authors:** Fatima Tarek, Najwa Hassou, Mohammed Nabil Benchekroun, Said Boughribil, Jamal Hafid, My Mustapha Ennaji

**Affiliations:** 1 Team of Virology and Oncology, Laboratory of Virology, Microbiology, Quality and Biotechnology/Ecotoxicology and Biodiversity, Faculty of Sciences and Techniques Mohammedia, University Hassan II of Casablanca; 2 Team of Biotechnology an Environment Laboratory of Virology, Microbiology, Quality and Biotechnology/ Eco toxicology and Biodiversity, Faculty of Sciences and techniques Mohammedia,University Hassan II of Casablanca; 3 Team of Eco toxicology and Biodiversity, Laboratory of Virology, Microbiology, Quality and Biotechnology/Ecotoxicology and Biodiversity, Faculty of Sciences and techniques Mohammedia, University Hassan II of Casablanca; 4 Team of Immuno parasitology, Laboratory food, Environment and Health FST Gueliz, University Cadi Ayyad Marrakech

**Keywords:** Carbon dioxide, hepatitis A virus, mutation rate, rotavirus, temperature variations

## Abstract

Enteric viruses are present in the environment as a result of the discharge of poorly or untreated wastewater. The spread of enteric viruses in the environment depend to human activities like stools of infected individuals ejected in the external environment can be transmitted by water sources and back to susceptible individuals for other cycles of illness. Among the enteric viruses Rotaviruses (RV) and Hepatitis A viruses (HAV) is the most detected in wastewater causing gastroenteritis and acute hepatitis. Therefore, it is of interest to climate change, mainly temperature and carbon Dioxide (CO2) variations, on Rotavirus and Hepatitis A as a model of enteric viruses present in the aquatic environment using computational modelling tools. The results of genetic ratio showed a negative correlation between the epidemiological data and the mutation rate. However, the correlation was positive between the temperature, CO2 increase, and the rate of mutation. The positive correlation is explained by the adaptation of the viruses to the climatic changes, the RNA polymerase of the RV induces errors to adapt to the environmental conditions. The simultaneous increase in number of infections and temperature in 2010 has been demonstrated in previous studies deducing that viral pathogenicity increase with temperature increase.

## Background

Rotaviruses (RV) are the most Common cause of diarrhea worldwide in children, rotavirus infections are associated to 200.000 deaths in children under 5 years of age in 2013 [1]-[2]. While hepatitis A virus (HAV) which is known as self-limiting disease, with high public health impact report about 1.300 new cases in 2014 [3-4. Rotavirus belongs to Reoviridae, it is a double stranded RNA, genus is divided into at least 7 genetic groups or geno groups (A-G). Genogroup A is the most involved in gastroenteritis pathogenicity for both Human and animals [5-6]. Different host species, interspecies transmissions and intra genic recombination are among the mechanisms responsible of genomic evolution of RV. Also, the accumulation of point mutations constantly in each RVs replication cycle leads to genetic draft [7-10]. And this is caused by the viral-encoded RNA-dependent RNA polymerase (RdRp) being error-prone [11].

Hepatitis A virus or HAV belong to Picornaviridae, spherical, about 30nm of diameter icosahedral capsid surrounding single stranded Monopartite, linear ssRNA(+) genome of 7.478 kb [12-14]. HAV has been initially classified in entero-virus genera in previously studies, although HAV has common characters with other genera of the picorna-virus family, it is significantly different and, present unique properties in relation to its genetic structure and replication procedure, that it is classified in hepato-virus genus as a sole species [15. HAV can infect Human and other primates, only one serotype and six different genetic groups, three isolated from Humans (I, II and III) and three from simian origin (IV, V, VI) have been described [16]. HAV mutation rate is significantly lower as compared to other members of the family Picornaviridae, and has an unusually small maximum genetic divergence [13. Liver is replication target and site of liberation of viral particles of HAV. Rotaviruses (RV) and Hepatitis A virus are transmitted mainly by fecal oral route. The contamination of the water represents the major cause of the spread of the virus in the environment. The surface runoff water is contaminated directly by discharge of none or undertreated wastewater or Human and animals swage in rivers or sea. While the underground water is contaminated through the soil by adsorption-desorption phenomenon [17-19]. Evolution and resistance of Rotaviruses (RV) and Hepatitis A virus (HAV) to different inactivation treatments are not depending only on errorprone nature of RV and HAV; also variations of climatic conditions have a major influence on genomic variation of viruses as a form of adaptation. It has been shown that variation of environment temperature have no effect on the prevalence of Rotaviruses; the infections linked to Rotaviruses were the same for all seasons, In winter as well as in summer, and also no correlation has been noticed for the other climatic factors such as rainfall, humidity or wind spread [20]. It is of interest to study the ratio between ratio between variation of rate of CO2 and Temperature, as climatic factors influencing on resistance of viruses in environment, and mutation rate on Rotaviruses (RV)and Hepatitis A virus (HAV) in different world areas for the period between 2000 and 2017.

## Methodology

For this study, data of infections by HAV and RV were collected from CDC Centers for Diseases Control and Prevention websites for 10 regions in the world, Latin America, Central Asia, Eastern Asia, Southeast Asia, Southern Asia, Western Asia, Oceania, Northern Africa and Sub-Saharan Africa during the period between 2000 and 2013 for Rotavirus (Table 1) and between 2004 and 2013 for Hepatitis A virus (Table 2). However, data is limited to countries where infections by HAV and RV are notified. Most data were from United Kingdom (UK) and United States of America (USA) mainly for HAV for the other areas; knowledge web literature was using. For climatic change, rate of global temperature and carbon dioxide, in this study we used data given by NASA Global Climate Change. Global surface temperature relative to 2000-2013 average temperatures (https://climate.nasa.gov/vitalsigns/ global- temperature/) and global distribution and variation of the concentration of carbon dioxide in parts per million (ppm) (https://climate.nasa.gov/vital-signs/carbon- dioxide/). Data for mutation rate has been collected from previous studies for Rotavirus of a period from 2005 to 2013 [21-24]. While mutation rate of Hepatitis A virus has been studied from database NCBI (National Center for Biotechnology Information) GenBank, 49 sequences of Hepatitis A virus collected was analyzed by MEGA software and mutation rate has been determined (Table 3). The results of mutation rate for both HAV and RV are shown in Table 4.

## Results and Discussion

**Figure 1:**
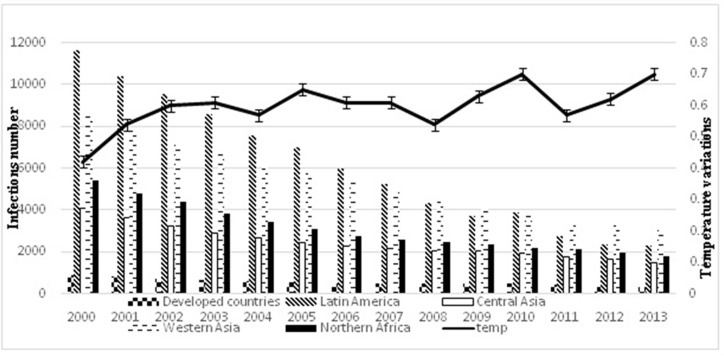
Effect of temperature variation on mutation rate of Rotavirus in studied geographical areas. Left: Coordinate axis for number of infections. Right: for temperature variation on °C. The curve shows that global temperature increases by time 0.4°C in 2000 and 0.7°C in 2013 for all studied areas.

**Figure 2:**
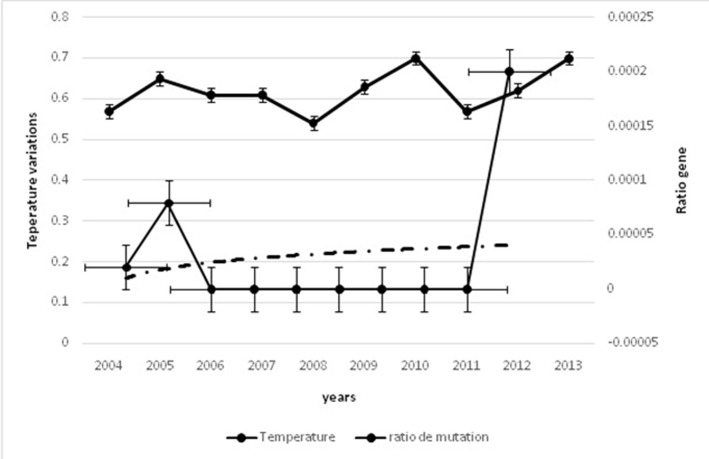
Correlation between temperature variation and mutation rate of Hepatitis A virus. The curve shows that most variation of temperature is important most rate of mutation of HAV increase.

**Figure 3:**
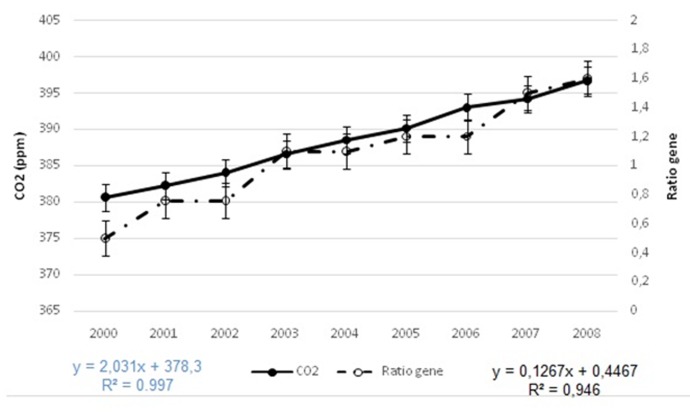
Correlation between CO2 and Rotavirus mutation ratio, CO2. Both curves are stackable, mutation ration of Rotavirus increase with increase of CO2 rate (at left CO2 rate, at right mutation rate).

**Figure 4:**
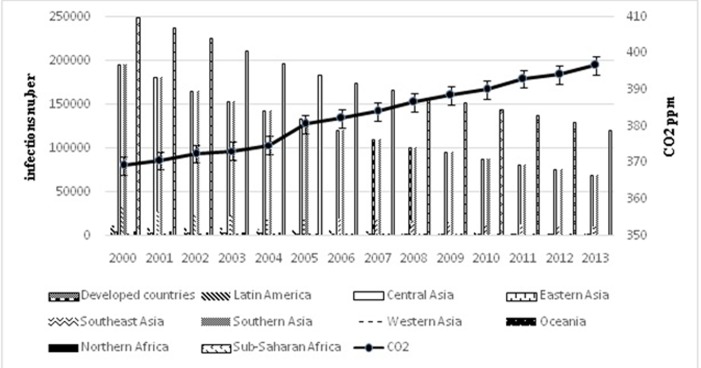
effect of CO2 on mutation rate of Rotavirus in 10 geographical areas. Left coordinate axis for rate of CO2 on ppm and right for number of infections. The curve shows that CO2 increase by time 370ppm in 2000 and 400ppm in 2013 for all studied areas. Developed countries have the higher rate.

**Figure 5:**
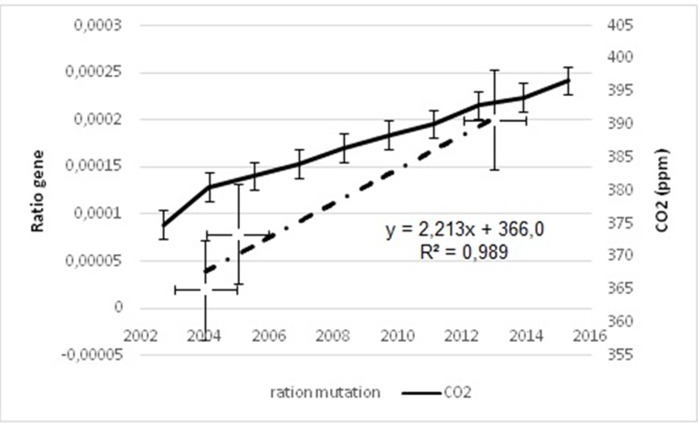
Correlation between CO2 and mutation rate of Hepatitis A virus. The curve shows that most rate of CO2 increase (from 375ppm in 2004 to 400ppm in 2014) most rate of mutation of HAV increase.

**Figure 6:**
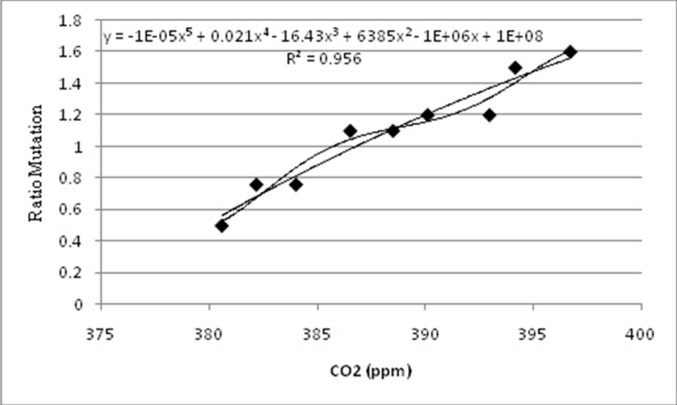
Linear regression and polynomial between CO2 and mutation rate of Rotavirus. Where the equation y = -1e-05 x5 + 0.0211 x4 -16.43 x3 + 6385 x2 - 1e+06 x + 1e+08 with R^2^ = 0.97 model the polynomial correlation among CO2 and mutation rate of Rotavirus. This is more representing than Linear equation (y=0.0619x - 22.975) with R2= 0.93

Results of data analysis have showed negative correlation between number of infections and change of temperature variations and CO2 rate. The positive correlation have been shown between temperature variations and mutation rate for both viruses Hepatitis A and Rotavirus (Figure 1 and Figure 2), also a positive correlation is shown between CO2 and mutation rate in all studied geographical areas (Figure 3 and Figure 4). For Rotavirus the curves of CO2 and mutation rate are stackable, the mutation rate increase with increase of CO2 (Figure 3). Variation of temperature and evolution of mutation rate are proportional for both studied viruses. For temperature variation a pick is shown in 2010 in all geographical areas. This study built a comprehensive database of RV and HAV, occurred between 2000 and 2013 in 10 geographical areas for RV and in UK and USA for HAV. Information about global temperature variation and carbon dioxide given by NASA has been also used. Analysis of these data shows a correlation between temperature variations, CO2 and mutation ratio of both viruses'RV and HAV (Figure 5). The analysis of the epidemiological profiles at the level of the developing and sub-Saharan countries and the climatic parameters (essentially CO2) shows an inverse relationship between the two parameters, whether at the level of the developed or sub-Saharan countries. Of the period between 2008 and 2012 a dive was observed in both populations but more intensive in the population of the developing countries and which cohere with a temperature increase of the earth's temperature. In order to better exploit this idea, we have to compare to the genomic level, whose mutation rate or the mutation ratio has almost the same speed and slope of the imitated amount of CO2 and then the deviation of CO2. According to our results, both viruses have the same slope, that mean that the mutation rate is the same for both RV and HAV viruses. Mutation production is not related to the characteristics of the virus itself, but it is a form of adaptation either to internalization or to resisting climate changes. Rotaviruses and HAVs are viruses that are present in the environment (release of Human waste into the external environment), the mutation rate increases for the entire genome of the virus including proteins adapting to environmental conditions [25]. It can be concluded that there is a strong correlation between climate change, including CO2 and temperature changes and mutation rate, which is mainly due to errors induced by RNA polymerase. The correlation between the three studied parameters (infection rate, temperature and ratio mutation) is well observed especially for the period between 2009 and 2011 with a peak in 2010, or a significant temperature values was recorded worldwide (developed and undeveloped countries), this massive increase in temperature (caused by CO2 increase) induced an increase in mutation ratio (Figure 6) and consequently increased pathogenicity for both RV and HAV viruses. Infections (epidemiological data given in Figure 1 and Figure 2) related to RV and HAV still show significant values despite medical and pharmaceutical efforts to develop vaccines to limit the occurrence of infections. Moreover, the climatic changes of temperature and CO2 are the major causes of appeared infections.

This is also confirmed by the relation established between the mutation rate and the deviation of CO2 at the level of the terrestrial envelope, with a linear regression of 93% whereas via a polynomial correlation can reach more than 97% as correlation with a logistic equation of the order of n = 4. The same results were observed for the HAV. All this allows us to conclude that there is a strong relationship between climate change and viral pathogenicity (Figure 6). In the same context, our results confirm previous studies that have demonstrated that the climate change likely affect the biology of the viruses' directly, because it is demonstrated that the higher temperature increase pathogen proliferation, we can explain that by the variation of mutations rate observed in our study that confirm that impact of climatic change on the pathogenicity is linked to the polymerase error [26]. However, the results have also shown a strong correlation between climate changes and increased viral pathogenicity and as a result, epidemics may emerge not only compared to Rotavirus and Hepatitis A virus but also to other RNA viruses. Therefore the effects of climate change must be taken particular account in development and monitoring programs. This study concerns two most interesting viruses for environmental virologists and explains the important numbers of pandemic and endemic events observed in Human and animal populations.

## Conclusion

Rotavirus causes the majority of viral gastroenteritis worldwide, while the Hepatitis A virus is implicated in acute viral Hepatitis. Rotavirus and Hepatitis A virus replicate in the enterocyte and hepatocyte respectively, and both are excreted by the faecal material and are subsequently released into the environment through the untreated wastewater. Viruses in their living environment are under the influence of several climatic factors. Temperature variations and CO2 rate are among the factors acting t on the living beings in the environment. The interaction between the two climatic factors studied and the behaviour of the Rotavirus and Hepatitis A genes had a positive correlation, whereas the increase of CO2 terrestrial and/or temperature induces an increase in mutation ratio of the viral RNA, these mutations are a form of adaptation to climate changes, in particular the variations in temperature and CO2 that the world experienced in the last few years as a result of pollution and the greenhouse effect. Viral infections pose a challenge despite the efforts made for the development of vaccines. This is due in fact to the genetic and molecular properties of RV and to maintain their survival.

## Conflict of Interest

Authors declare no conflict of interest

## Figures and Tables

**Table 1:** Epidemiological data of Rotaviruses infections in 10 geographical areas, for period between 2000 and 2013

Year	2000	2001	2002	2003	2004	2005	2006	2007	2008	2009	2010	2011	2012	2013	Moy
Developed countries	903	815	741	674	616	562	523	486	479	487	484	464	354	336	566
Latin America	11631	10382	9536	8612	7543	7021	6011	5231	4355	3718	3903	2747	2383	2288	6097,21
Central Asia	4106	3616	3233	2912	2670	2463	2299	2158	2058	2053	1929	1790	1650	1504	2460,07
Eastern Asia	195807	181661	165884	153585	142606	133529	120985	109894	101679	95314	88547	81748	75641	70109	122642,07
Southeast Asia	32263	29183	26531	24112	22214	20481	18980	17771	16338	15027	13931	12760	11567	10765	19423,07
Southern Asia	195807	181661	165884	153585	142606	133529	120985	109894	101679	95314	88547	81748	75641	70109	122642,07
Western Asia	8566	7796	7278	6852	6130	5770	5383	4833	4460	4077	3715	3446	3331	3143	5341,43
Oceania	594	596	613	576	504	553	526	524	515	462	491	483	446	414	521,21
Northern Africa	5426	4804	4375	3855	3449	3081	2763	2605	2502	2346	2213	2136	1957	1792	3093,14
Sub-Saharan Africa	249612	237746	225705	210837	196757	183953	174133	166477	158084	152045	145022	137913	129794	121009	177791,93
Total	527984	493603	458550	424350	392868	366193	339232	316587	296266	280737	264862	247632	230843	214806	346750,93

**Table 2:** Epidemiology of Hepatitis A virus (HAV) in United Kingdom (UK) and United States of America (USA), variation of global temperature, CO_2_ and mutation rate between 2004 and 2013

	2004	2005	2006	2007	2008	2009	2010	2011	2012	2013
UK	753	632	617	605	623	623	403	492	454	477
USA	4488	3579	2979	2585	1987	1670	1398	1562	1781	1239
T (°C)	0.57	0.65	0.61	0.61	0.54	0.63	0.7	0.57	0.62	0.7
CO_2_(ppm)	374.88	380.62	382.22	384.05	386.56	388.54	390.14	393	394.19	396.74
Ratio Mutation (10^-3^)	0.04	0.04	0.04	0.1	0.1	0.14	0.14	0.14	0.14	0.14

**Table 3:** Hepatitis A virus sequences collected from database NCBI (National Center for Biotechnology Information) GenBank and used for determination of mutation rate by Mega Software

Sequence Code	Year	Nomination
KX035096.1	2013	Hepatovirus A isolate 18f.1 complete genome
JQ425480.1	2012	Hepatitis A virus strain HAS-15 complete genome
AB793726.1	2012	Hepatitis A virus gene for polyprotein complete cds isolate:
AB793725.1	2012	Hepatitis A virus gene for polyprotein complete cds isolate:
KF569906.1	2012	Hepatitis A virus strain DH01 complete genome
KT877158.1	2012	Tupaiahepatovirus A isolate TN1 complete genome
NC028981.1	2012	Tupaiahepatovirus A isolate TN1 complete genome
LC049342.1	2012	Hepatovirus A genomic RNA complete genome isolate: MNA06-2148
LC049341.1	2012	Hepatovirus A genomic RNA complete genome isolate: MNA12-130
LC049337.1	2012	Hepatovirus A genomic RNA complete genome isolate: MNA12-001
JQ655151.1	2011	Hepatitis A virus isolate Kor-HAV-F complete genome
AB819870.1	2011	Hepatitis A virus gene for polyprotein complete cds isolate:
AB819869.1	2011	Hepatitis A virus gene for polyprotein complete cds isolate:
AB909123.1	2011	Hepatitis A virus genomic RNA nearly complete genome isolate:
KY003229.1	2011	Hepatovirus A complete genome HA12-0938
LC049340.1	2010	Hepatovirus A genomic RNA complete genome isolate: MNA10-B1355 HA12-0796
KT819575.1	2010	Hepatovirus A isolate KibOB-1 complete genome
KC182587.1	2009	Hepatitis A virus isolate A2 complete genome HAJFF-Kan12
KC182588.1	2009	Hepatitis A virus isolate B1 complete genome HAJTS-SinKan11
KC182589.1	2009	Hepatitis A virus isolate A3 completegenome
LC049338.1	2009	Hepatovirus A genomic RNA complete genome isolate: MNA09-B1141 HAJHM-PapTok11
AB839696.1	2007	Hepatitis A virus genomic RNA complete genome isolate: SoloA07-P15
AB839695.1	2007	Hepatitis A virus genomic RNA completegenomeisolate: MataramA07-RS03
AB839694.1	2007	Hepatitis A virus genomic RNA complete genome isolate: MakassarA07-R18
AB839693.1	2007	Hepatitis A virus genomic RNA complete genome isolate: JemberA07-SBY07
LC049339.1	2006	Hepatovirus A genomic RNA complete genome isolate: MNA06-2130
AF485328.1	2003	Hepatitis A virus isolate LY6 complete genome
AB839692.1	2003	Hepatitis A virus genomic RNA complete genome isolate: BaliA03-H29
AB839692.1	2003	Hepatitis A virus genomic RNA complete genome isolate: BaliA03-H29
HV192265.1	2000	JP 2000512841-A/1: Simian-human HAV having a chimeric 2C protein
LC128713.1	2000	Hepatovirus A genomic RNA nearly complete genome strain: Banglane2000
AB618531.1	1999	Hepatitis A virus genomic RNA complete genome isolate: HAJNS-BorSap10
M59810.1	1993	Hepatitis A virus polyprotein RNA completecds
KX523680.1	1988	Hepatovirus A isolate LV8 complete genome
M20273.	1986	Human hepatitis virus type A RNA complete genome
K02990.1	1985	Human hepatitis A virus complete genome
HQ246217.1	1980	Hepatitis A virus strain CFH-HAV complete genome
AB623053.1	1957	Hepatitis A virus genomic RNA nearly complete genome isolate:
KP879216.1	2015	Hepatitis A virus isolate 18f complete genome
LC191189.1		Hepatovirus A genomic RNA complete genome isolate: HA16-0511
KX088647.1		Hepatovirus A isolate HM175-HP polyproteingenecompletecds
KT229612.1		Hepatovirus A isolate 3ID complete genome
KT229611.1		Hepatovirus A isolate 2ID complete genome
KF724017.1		Hepatitis A virus isolate L0 polyproteingenecompletecds
KF724018.1		Hepatitis A virus isolate F0.05A polyproteingenecompletecds
KF724019.1		Hepatitis A virus isolate F0.05LA polyproteingenecompletecds
KF724020.1		Hepatitis A virus isolate F0.2A polyproteingenecompletecds
KF724021.1		Hepatitis A virus isolate F0.2LA polyproteingenecompletecds
KF724022.1		Hepatitis A virus isolate R0.05A polyproteingenecompletecds HA286-Aki1957
KF724023.1		Hepatitis A virus isolate R0A polyproteingenecompletecds
KF773842.1		Hepatitis A virus isolate 112572/2013 polyproteingenecompletecds

**Table 4:** Temperature variations (°C), rate of CO_2_ (ppm) and mutation rate of HAV and RV between 2000 and 2013

		2000	2001	2002	2003	2004	2005	2006	2007	2008	2009	2010	2011	2012	2013
CO_2_ (ppm)		369.29	370.59	372.53	373.2	374.88	380.62	382.22	384.05	386.56	388.54	390.14	393	394.19	396.74
Temperature variation (°C)	HAV	0.42	0.54	0.6	0.61	0.57	0.65	0.61	0.61	0.54	0.63	0.7	0.57	0.62	0.7
Ratio mutation (10^-3^)	RV	0.04	0.04	0.04	0.04	0.04	0.04	0.04	0.1	0.1	0.14	0.14	0.14	0.14	0.14
			-	-	-	-	0.5	0.76	0.76	1.1	1.1	1.2	1.2	1.5	1.6
